# Comparison of SVM and ANFIS for Snore Related Sounds Classification by Using the Largest Lyapunov Exponent and Entropy

**DOI:** 10.1155/2013/238937

**Published:** 2013-09-30

**Authors:** Haydar Ankışhan, Derya Yılmaz

**Affiliations:** ^1^Department of Biomedical Equipment Technology, Vocational School of Technology, Başkent University, 06810 Ankara, Turkey; ^2^Department of Electrical and Electronic Engineering, Faculty of Engineering, Başkent University, 06810 Ankara, Turkey

## Abstract

Snoring, which may be decisive for many diseases, is an important indicator especially for sleep disorders. In recent years, many studies have been performed on the snore related sounds (SRSs) due to producing useful results for detection of sleep apnea/hypopnea syndrome (SAHS). The first important step of these studies is the detection of snore from SRSs by using different time and frequency domain features. The SRSs have a complex nature that is originated from several physiological and physical conditions. The nonlinear characteristics of SRSs can be examined with chaos theory methods which are widely used to evaluate the biomedical signals and systems, recently. The aim of this study is to classify the SRSs as snore/breathing/silence by using the largest Lyapunov exponent (LLE) and entropy with multiclass support vector machines (SVMs) and adaptive network fuzzy inference system (ANFIS). Two different experiments were performed for different training and test data sets. Experimental results show that the multiclass SVMs can produce the better classification results than ANFIS with used nonlinear quantities. Additionally, these nonlinear features are carrying meaningful information for classifying SRSs and are able to be used for diagnosis of sleep disorders such as SAHS.

## 1. Introduction

Sleep apnea/hypopnea syndrome (SAHS) is a sleep disorder which is defined by instances of low-level breathing or pauses in breathing during sleep. SAHS is diagnosed by polysomnography (PSG). However, PSG is highly troublesome test owing to the patients sleeping for an overnight in the hospital. Researchers indicate that there is a serious requirement to simplified systems instead of PSG for diagnosing SAHS [1]. For this purpose, some approaches have been developed by using several physiological signals such as electrocardiography (ECG), electroencephalography (EEG), and snore related sounds (SRSs). Recently, there are many studies available for diagnosing SAHS working with the SRSs [2–9].

The snoring is one of the SAHS's symptoms, and it may be interpreted as an indicator for many different diseases such as cardiovascular and sleep disorders [10]. It is stated that snoring is associated with physical and functional defects of upper airway responses and generally occurring in patients having narrower upper airway than the normal ones [3, 4, 6, 11]. Hence, the knowledge about physiology and functions of upper airway can be obtained from SRSs [4]. Because SAHS is concerned with partial or full collapse of upper airways, many studies using different linear techniques have been performed for SRSs analysis [5, 7, 12–16]. The results of these studies indicate that SRSs carry significant information on SAHS.

Recently, some studies have been shown to classify SRSs that is a first task for detecting snoring and SAHS [17–20]. Duckitt et al. [17] presented a method which provides the automatic monitoring of snoring characteristic depending on intensity and frequency of data. They used mel-frequency cepstral coefficients (MFCCs) with hidden Markov model (HMM). Their system obtained 82–89% accuracy in specifying snores. Cavusoglu et al. [18] estimated energy and zero-crossing rates of SRSs and used linear regression method for classifying SRSs' segments as snore/nonsnore. They obtained 86.8% accuracy for obstructive sleep apnea (OSA) patients. Karunajeewa et al. [19] calculated zero-crossing rates, normalized autocorrelation coefficients, energy, and first predictor coefficient of linear predictive coding (LPC) analysis as features for classification of SRSs into snoring, breathing, and silence. They also used three noise reduction techniques and compared their performance. While they had an overall classification accuracy of 90.74%, their accuracy is up to 96.78% with noise reduction techniques and a proper choice of features. Yadollahi and Moussavi [20] tried to classify breath and snore sounds by using Fisher linear discriminant (FLD) based on zero-crossing rates, energy, and first formant of the SRSs that are recorded by two microphones (tracheal and ambient). They investigated the body and neck positions' effects on the classification results. The overall accuracies were found as 95.7% and 93.2% for tracheal and ambient recordings regardless of the neck position, respectively [20].

It is seen that these studies have focused on examining snoring sounds by linear features analysis such as Fourier transform, LPC, but the nonlinear characteristics of SRSs for classifications have not yet been evaluated so far. SRSs are known to have nonstationary and complex behaviours [21, 22]. Some parts of SRSs have nonlinear acoustic vibrations depending on out- and in-factors such as the respiratory airflow strength, vibrations on the soft palate, the shape and differences of upper airway, and the airway obstruction due to tongue subsidence [22, 23]. These make it difficult to solve the identification of SRSs depending on traditional linear frequency and time domain based methods. In these cases, the nonlinear analysis can be performed to reveal the undefined characteristics of the signal. It is possible to use the linear and nonlinear analyses together since these approximations can produce the meaningful results which support each other. For example, a wideband spectrum may be indicator of chaos, but it cannot only identify definition of chaos. However, spectral analysis may give some information for examining the source of variations in the signals.

From the biomedical perspective, nonlinear analyses methods coming from chaos theory have been used to evaluate the states of biological systems and signals from systemic level to the cellular scale [24–28]. The behaviours of dynamical biological systems are interpreted by evaluating the chaotic indicators such as Lyapunov exponents, correlation dimension, Poincare section, and entropy. While some systems show a chaotic behaviour under the normal operating conditions, at the pathological conditions a degree of systems' chaotic behaviour may be reduced or increased, or system behaviour can turn to a regular state [23–28]. Hence, some contributions related to commentating of system functions can be provided by evaluating the effects of pathological or normal conditions on the system behaviour. There are a few studies which investigated the nonlinear properties of SRSs in the literature [29–33]. Sakakura [29] used Poincare section and the largest Lyapunov exponent (LLE) for indicating the chaotic characteristics of snoring sounds. Mikami [30] performed surrogate analysis by using correlation integral and showed that snoring sounds have nonlinear properties. Yılmaz and Ankışhan [31] calculated the LLE values for snore segments obtained from apnea/hypopnea patients and simple snorers. Their results showed that the LLE of simple snorer group was significantly higher than apnea/hypopnea group [31]. Ankışhan and Arı [32] showed from their experimental results that SRSs have chaotic behaviour and carry meaningful information, so the LLE can be used for classification of SRSs. Moreover, they also showed that entropy can be used in classification of SRSs with time domain features [33]. These studies have shown that nonlinear measures can bring to light significant outcomes for SRSs.

The LLE quantifies the dynamic stability of a system and gives information about the predictability of a system [34, 35]. The positive value of the LLE is accepted as a key indicator of chaotic behaviour. Entropy which has also multiple definitions may be interpreted as the average amount of new information obtained by making the measurement in information theory sense [35]. In previous studies, it was reported that these features are the quantitative measures of chaos and frequently used in the nonlinear analysis of the experimental time series [23–33]. So, the aims of this work are to extract meaningful information from SRSs related to its nonlinear characteristics, to investigate the variability of these chaotic measures for different SRSs segments, and to test the availability of these quantities for classifying SRSs. For this purpose, SRSs were analysed by using entropy and the LLE and classified as snore/breathing/silence with support vector machines (SVMs) and adaptive network fuzzy inference system (ANFIS).

There have been several methods which are used for classification of biological data. Two of them are ANFIS and SVMs which are effective methods for classification and machine learning systems such as face detection, speech recognition, pattern recognition and lots of biomedical applications [36–41]. Recently, multiclass SVMs have been very popular and successful for different classification issues [42, 43]. In previous studies, for classifying SRSs, some classification methods such as the linear regression, LPC, HMM, and FLD have been used [17–20]. Unlike the previous ones, in this study, it has been taken into consideration the successfulness of SVMs and ANFIS in biomedical applications; the multiclass SVMs and ANFIS were chosen as classifiers.

## 2. Material and Methods

### 2.1. Data

In this study, Cavusoglu et al.'s [18] data set, obtained from Gülhane Military Medical Science Sleep Laboratory, was used. While recording data, a Sennheiser ME 64 condenser microphone with a 40 Hz–20 kHz ± 2.5 dB frequency response was used and placed 15 cm over the patient's head. So, a BNC cable, UA-1000 model multichannel data acquisition system, and a personal computer were used for obtaining the data. The computer was placed out of the room for avoiding its noise. Signal records were made at 16 kHz sampling frequency and 16-bit resolution and stored with patient information. The details about this database are referred to in Cavusoglu et al.'s study [18]. 12 patients' data were used in the experiments of this study. The information of related patients is given in Table 1.

### 2.2. Entropy

Entropy is defined in terms of a random variable's probability distribution and generally interpreted as a measure of uncertainty. The concept of entropy is used in very large area, from physic to information theory [35]. The amount of entropy is often thought of as the amount of information used to define the system's state [35]. Shannon gives the definition of entropy which states the content of expected information or uncertainty of a probability distribution [44]. In this work, with the considering successfulness about the short signal entropy estimation, Shannon entropy algorithm was used to evaluate the average amount of new information obtained from segments of SRSs [44]. Shannon proposed a quantity called self-information which is a logarithmic function on the interval (0,1] and related to the probability of event *X* = *x*_*i*_, (*i* = 1,2,…, *n*). Self-information *h*(*p*_*i*_) is given by

(1) $$\begin{array}{c} h\left(p_{i}\right)=\text{lo}{\text{g}}_{2}\left(\frac{1}{p_{i}}\right). \end{array}$$

Average self-information or entropy is derived by weighing the *n* numbers of self-information values *h*(*p*_*i*_) by their individual probabilities [44]:

(2) $$\begin{array}{c} H_{k}=\overset{n}{\underset{i=1}{\sum }}p_{i}\text{lo}{\text{g}}_{2}\left(\frac{1}{p_{i}}\right). \end{array}$$

### 2.3. Phase Space Reconstruction

The concepts coming from nonlinear dynamic theory are applied to biomedical time series by using some methods proposed for calculating the chaotic measures (Lyapunov exponents, correlation dimension, etc.). For estimation of these measures, the first step is the reconstruction of system's attractor (phase space reconstruction) from the scalar time series data [34, 35]. Even though the dynamical system might have many variables, the obtaining of one variable from the system is sufficient for reconstructing the phase space of the system [35]. The time-delay technique is easy, systematic, and the most popular approach to reconstruct the attractor [34, 35]. In this study, Takens' time-delay embedding method [45] was used for the reconstruction of the attractor from SRSs segment parts in the phase space. According to embedding theorem of Takens [45], *m*-dimensional time-delay vectors are reconstructed from SRS segment parts (*x*(*n*) = *x*(*n*Δ*t*), *n* = 1,2,…, *N*), as follows:

(3) $$\begin{array}{c} X_{i}=\left(x_{i},x_{i+\tau },x_{i+2\tau },x_{i+3\tau },\ldots ,x_{i+(m-1)\tau }\right), \end{array}$$

where *x*(*i*) is the *i*th value of the sound segment part, *X*_*i*_ is the *i*th point on the attractor (*i* = *N* − (*m* − 1)*τ*), *m* is the dimension of the phase space (embedding dimension), and *τ* is the lag or time delay [45]. In this theorem, selection criterion is *m* ≥ 2*D* + 1 for embedding dimension; herein, *D* is the attractor dimension. According to Abarbanel [34], *m* > *D* would be sufficient. The chosen embedding dimension and time-delay parameters are important and determined from time series data, since the optimal values of them are not known in advance. There are many proposed methods for choosing their values [35]. In this paper, the false nearest neighbours (FNN) method was applied to select the embedding dimension [46]. The suitable embedding dimension is determined as the phase space dimension for the zero percentage value of FNN where the number of FNN is below a threshold value. The average mutual information (AMI), that is, a nonlinear autocorrelation function, was used for finding the time delay [34, 47]. In this study, the proper value of time delay is selected as the first minimum value of AMI.

### 2.4. The Largest Lyapunov Exponent

The Lyapunov exponents or characteristic exponents specify the sensitivity to initial conditions of a dynamical system and measure the average rate of convergence or divergence of nearby trajectories on the attractor [34, 35, 48]. For detection of the chaotic behaviour, the calculation of the LLE is sufficient since a positive LLE can be accepted as good evidence for a chaotic system [34, 35, 48]. In this study, the LLE measure was used in the determination of the complexity of the SRS segments.

Several methods estimating the LLE or Lyapunov exponent spectrum have been proposed [48–51]. A simple method which calculates only the LLE is Rosenstein et al.'s algorithm [51], and this algorithm is suitable especially for small noisy datasets. In this paper, Rosenstein et al.'s algorithm [51] was chosen to calculate the LLE of snore episodes. It was stated that this algorithm is a suitable method for embedding dimension is smaller than Takens' selection criterion and for a wide range of time delays [51].

According to Rosenstein et al.'s algorithm [51], after reconstructing the time-delay vectors, the nearest neighbours of each state on the phase space trajectory are searched. It is opined that the *j*th nearest neighbours' pair diverges nearly at a rate dedicated by the LLE (*λ*_1_) [51]:

(4) $$\begin{array}{c} d_{j}\left(i\right)\approx C_{j}e^{\lambda_{1}(i.\Delta t)}, \end{array}$$

where *C*_*j*_ is the initial separation, Δ*t* is the sampling period, and *d*_*j*_(*i*) is the distance between the *j*th pair of nearest neighbours after (*i* × Δ*t*) seconds. Equation (5) is obtained by taking the logarithm of both sides of (4):

(5) $$\begin{array}{c} \ln d_{j}\left(i\right)\approx \ln C_{j}+\left(i\cdot \Delta t\right). \end{array}$$

This equation gives a set of approximately parallel lines (for *j* = 1,2,…, *M*), each with a slope roughly proportional to *λ*_1_. The LLE values of sound segment parts are computed by using a least squares fit to the averaging line defined by

(6) $$\begin{array}{c} y\left(i\right)=\frac{1}{\Delta t}\langle \ln d_{j}\left(i\right)\rangle , \end{array}$$

where 〈⋯〉 represents the average of all *j* values [51].

### 2.5. Support Vector Machines

Support vector machines (SVMs) is a tool used in machine learning. It was proposed by Cortes and Vapnik in 1995 [52]. SVMs that motivate minimizing Vapnik-Chervonenkis (VC) dimension have proved to be very successful in classification learning [52–54]. In this algorithm, it is easy to formulate the decision functions in terms of a symmetric, positive definite and square integrable function *k*(·, ·) referred to as a kernel. The class of the decision functions also known as kernel classifiers [55] is then given by

(7) $$\begin{array}{c} f\left(x\right)=\mathrm{sign}\left(\overset{l}{\underset{i=1}{\sum }}\alpha_{i}y_{i}k\left(x_{i},x\right)\right),\;\alpha \geq 0, \end{array}$$

where training data *x*_*i*_ ∈ *R* ^*d*^ and labels *y*_*i*_ ∈ {±1}.

#### 2.5.1. Multiclass Support Vector Machines

SVM has been used in numerous studies of interest in different areas. When SVMs are performed for multiclass problems, the generally used technique is to solve the problem as a collection of some two-class classifications that can be solved by binary SVMs [54].

According to Vapnik [54], some information is stated as follows: suppose that we have a set of training data ((*x*_*i*_, *y*_*i*_), for *i* = 1,…, *n* and *x*_*i*_ ∈ *R* ^*d*^ and *y*_*i*_ ∈ {±1}) and a nonlinear transformation to a higher dimensional space, the feature space $\left(\Phi (\cdot ,\cdot ),R^{d}\overset{\Phi (\cdot ,\cdot )}{\to }R^{H}\right)$, the SVMs solves

(8)  Minimize(w,ζi,b)⁡ {12||w||2+C∑iζi}

(9) Subject  to   yi(ϕT(xi)w+b)≥1−ζi,∀i=1,…,n, ζi≥0,

where (*w*, *b*) defines the linear classifier in the feature space. The SVMs try to enforce positive samples to present an output greater than +1, and the negative samples present an output less than −1 for two classes. Those samples are not fulfilling this condition and need a nonzero *ζ*_*i*_ in (9), so they will introduce a penalty in the objective function (8). The inclusion of the norm of *w* in (8) checks whether the solution is maximum margin [54].

The purpose of *C*∑_*i*_ *ζ*_*i*_ is to control the number of misclassified samples. The usage of a larger value of parameter *C*, which is chosen by the user, corresponds to assigning a higher penalty to errors. When application of this optimization for solving problems, generally suffering the balance of the samples between the classes and the unequal density of the clusters in the feature space [56]. In multiclass support vector machines, the binary SVMs, that is, using a vector of different weight and bias for each class (*w* ^*j*^ and *b* ^*j*^ for *j* ∈ {1,2,…, *k*}), are applied. The classifier function of SVMs is given as

(10) $$\begin{array}{c} f\left(x\right)=\arg\cdot \max\left(\phi^{T}\left(x\right)w^{j}+b^{j}\right),\;j\in \left\{1,\ldots ,k\right\}. \end{array}$$

Accordingly, we can impose (9) for each class it does not belong to, as suggested in [54, 57], leading to

(11)  Minimize(wj,bj,ζij,m)⁡ {12∑j=1k||wj||2+C∑j=1k ∑m=1m≠jk ∑i=1njζij,m},Subject  to (ϕT(xij)wj+bj)−(ϕT(xij)wm+bm)≥2−ζij,m, ζij,m≥0,  ∀j=1,…,k,∀m=1,…,k(m≠j),  ∀i=1,…,nj,

where *x*_*ij*_ is the *i*th sample in class *j* and *n*_*j*_ is the number of training samples in that class. Herein, this optimization problem searching the *j*th output for *x*_*ij*_ is larger than any other. In conclusion, the penalization for any incorrectly classified sample will be based on the incorrectly assigned class and on the number of outputs larger than the output of true assigned class [57]. We have focused our study on that this simple model can be extended to solve nonlinear separable problem. So, there are some studies available in the literature, which have proposed to use kernel-based methods. These methods used mapping functions on the input features for carrying them into a very high-dimensional space. So, the methods can construct a hyperplane in that feature space properties rely on kernel functions of SVMs [54, 58].

### 2.6. Adaptive Network Fuzzy Inference System (ANFIS)

Adaptive network fuzzy inference system (ANFIS) is a tool for using different purposes. Its structure has a fuzzy set “*if-then rules.*” For generating the suitable input-output pairs, it has a correct membership function [59]. ANFIS has a learning ability having a neuro-fuzzy-type structure, and this network have nodes, where each combination of these nodes placed in the different layers for completed specific functions.

ANFIS has some hybrid learning algorithm, that is, Sugeno-type fuzzy inference systems for identifying parameters. Sugeno-type fuzzy system uses least-squares method which is combined for training membership function parameters of fuzzy system. This system emulates a given training data set [59].

Sugeno-type ANFIS model has five layers for generating inference system [59]. There are several nodes in each layer. These layers are working as follows: the input signals in the present layer are the output signals obtained from nodes in the previous layers [59]. Jang [59] has shown that first-order Sugeno-type model is given as

**Figure 1:**
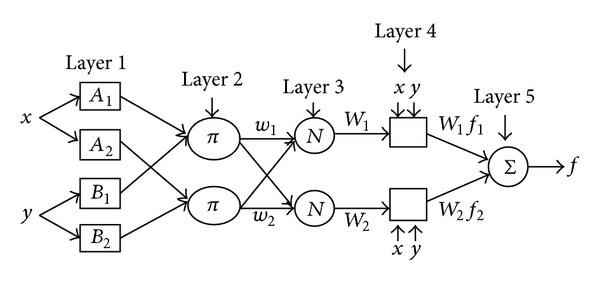
Sugeno-type adaptive network fuzzy system [59].

(12) Rule  1:  if  (x  is  A1),  (y  is  B1),  then  (f1=p1x+q1y+r1),Rule  2:  if  (x  is  A2),  (y  is  B2),  then  (f2=p2x+q2y+r2),

where *x*_1,2_ are inputs, *A*_*i*_ and *B*_*i*_ are fuzzy sets, *f*_*i*_ outputs within the fuzzy region identified by the rules, and the design parameters (*p*_*i*_, *q*_*i*_, and *r*_*i*_) are determined during the training process [59]. ANFIS architecture is given in Figure 1.

In Figure 1, square nodes which are called adaptive nodes are accepted to represent the parameter sets in these nodes which are adjustable [59]. However, circle nodes are fixed nodes that are accepted to represent the fixed parameter sets in the system [59]. The details of ANFIS model can be seen in [59].

## 3. Results

### 3.1. Data Analysis and Feature Extraction

**Figure 2:**
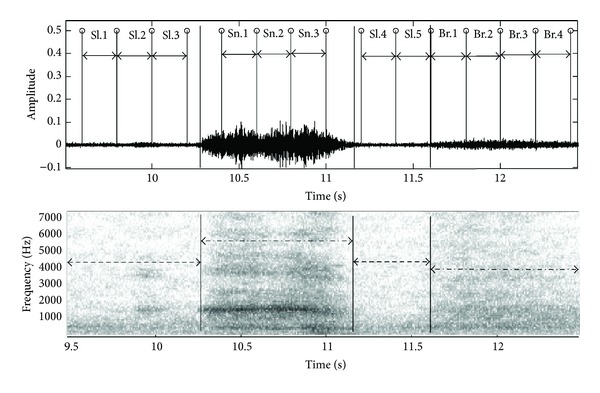
Subdivision procedure of SRSs segments: snore, breathing, and silent segment parts.

In this study, snore, breathing, and silent segments were firstly extracted from the SRSs. The samples of the recorded ambient sounds in time and time-frequency domains are shown in Figure 2. Herein, snore, breathing and silent segments were manually marked in both domains for examining the signals' characteristics. Each segment was automatically subdivided into parts having 3200 samples by developed MATLAB program with thin black lines. These segmentation and subdivision procedure were also shown in Figure 2. In Figure 2, Br.1, Br.2,…, and Br.4 represent breathing segment parts, Sn.1,…, and Sn.3 represent snore segment parts, and Sl.1, Sl.2,…, and Sl.5 represent silent segment parts. We can see from Figure 2, time duration of each segment (in case of inspiration and expiration interval) is different from each other. The frequency and amplitude spectrums of SRSs segments have different characteristics (in Figure 2). It is seen that snore segment parts have a few components for different frequency range as high and low amplitude peaks in Figure 2. However, breathing and silent segments have many components which are similar peak values in a wide frequency band. The separation of breathing and silent segments is difficult since their characteristics are similar to each other, and the noise originated from ambient recordings substantially affects breathing and silent segments. Therefore, auditory and visual examinations were used for marking of the segments.

In case of experiments, the segments were normalized to be in the range of [−0.5,0.5]. This method is used because of each subject having different characteristics (i.e., all snore, breathing, and silent segments' amplitudes of subjects are different owing to their sounds intensity). Therefore, changing range of each sound segment could be different without normalization. It would affect the calculated feature values.

**Figure 3:**
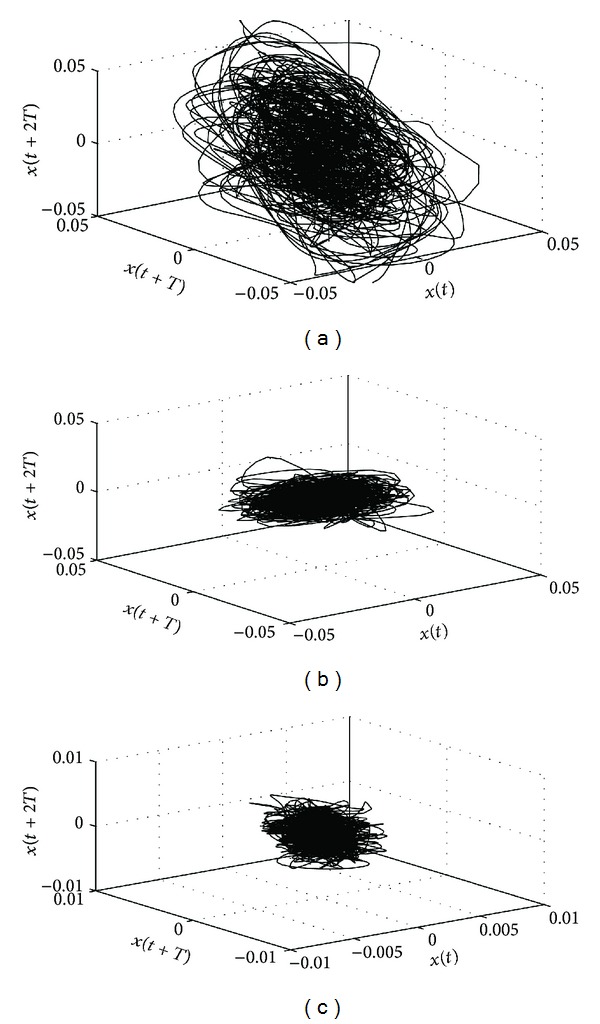
Three reconstructed SRSs' segment attractors with *m* = 3 and *T* = *τ* = 8: (a) snore, (b) breathing, and (c) silent segment attractors.

In order to investigate the chaotic behaviour of SRSs, LLE and entropy of each segment parts are calculated. For the LLE calculation, all segment parts are reconstructed in the phase space according to Takens' theorem. Therefore, embedding dimension (*m*) and time delay (*τ*) values must be determined, firstly. In order to make a decision about these values, for all SRS segments, embedding dimension (*m*) and time delay (*τ*) values are estimated by using the false nearest neighbour (FNN) and average mutual information (AMI) methods, respectively. Looking at these results, it was seen that *m* and *τ* values of snore, breathing and silent segment parts are close to each other. Hence, if we chose *m* to be 5 and *τ* to be 8 for all segment parts, the estimation of LLE is not significantly affected. So, it is most suitable to set *m* = 5 and *τ* = 8 for all segment parts. Herein, some reconstructed attractors depending on SRSs chosen segments projected onto three-dimensional phase space are shown in Figure 3. Certain type of geometrical shape of snore, breathing and silent segments can be seen from the figure. Moreover, sizes of attractors give information to us about how they are differentiated from each other depending on the amplitude values of SRS segments.

**Figure 4:**
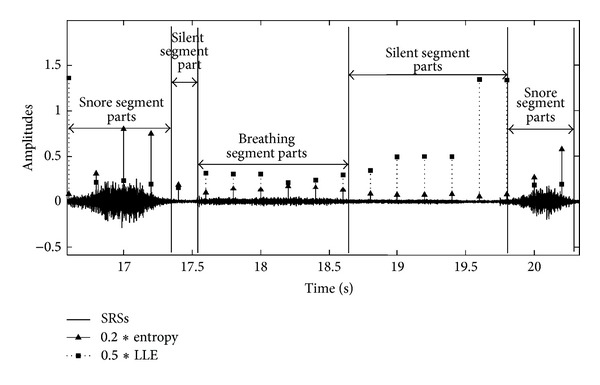
The LLE and entropy values of SRSs segments.

Figure 4 gives information about LLE and entropy features of SRS segments. The graph is manually divided and marked with vertical lines to show the segments. The LLE and entropy values of segment parts (square and triangle markers, resp.) are automatically estimated by developed MATLAB program.

**Figure 5:**
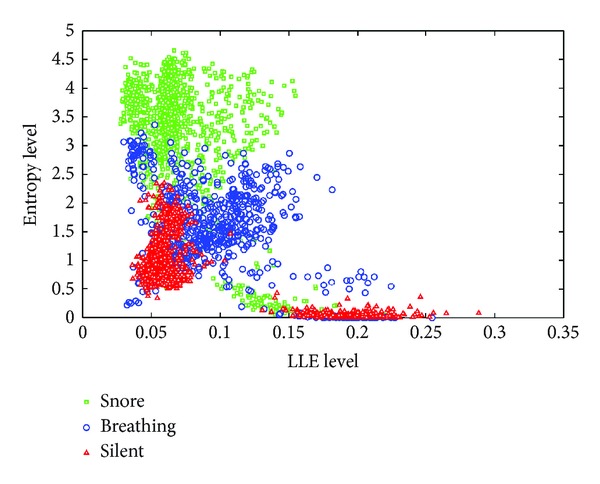
Distribution of data sets in LLE versus entropy.

It can be seen from Figure 4 that the LLE and entropy values can be differentiated into snore, breathing, and silent segments. For snore segments, LLE values changing between (0.1 × 2) and (1.2 × 2) are less than breathing and silent ones, and entropy values changing between (0.1 × 5) and (1 × 5) are greater than breathing and silent ones. However, at the breathing segments, entropy values are usually greater and LLE values are usually less than silent segments. When looking at silent segments, they generally have bigger values of LLE and smaller values of entropy.  Figure 5 gives us any information about all snore/breathing/silent parts distribution in all data sets.

In Figure 5, snore, breathing, and silent segment parts of SRSs are denoted by square, circle, and triangle symbols, respectively. It is seen that snoring segment parts are usually clustered between 2.0 and 4.5 entropy levels and 0.03–0.15 LLE levels; however, some parts of snoring segments are clustered between 0.0 and 1.0 entropy levels and 0.1–0.15 LLE levels. Although snoring segment parts are clustered into two areas, the results show they can be discriminated from breathing and silent segment parts. If looking at the breathing segment parts, which are usually clustered between 0.0 and 3.3 entropy levels and 0.03–0.25 LLE levels. The results show that breathing segment parts cannot easily be classified depending on the distribution area. Because feature distribution of breathing parts is not significantly clustered. However, silent segment parts are generally clustered into two areas: first, between 0.0 and 0.3 entropy levels and 0.15–0.25 LLE levels; second, between 0.3 and 2.5 entropy levels and 0.4–1.0 LLE levels. It can be said that silent segment parts can be discriminated and classified into their own classes.

### 3.2. Experiments

In this study, the performance of methods in classifying snore, breathing, and silent segments' parts were evaluated in two experimental ways.

*Experiment I.* The training and test datasets were obtained from the recordings of same patients. The first half interval of the recordings was training data set and the rest was test data set. These datasets obtained from 12 patients include 672 and 668 segments for training and test, respectively. After the subdivision procedure of these segments, training and test databases have 4037 and 5234 segment parts, respectively.

*Experiment II.* The training and test data sets were obtained from all recordings of different patients. Training data set contains 553 segments from 6 patients, and the rest 6 patients recordings were used for test data set with 787 segments. After the subdivision procedure of these segments, training and test databases have 3322 and 4110 segment parts, respectively.

The details of the training and test datasets in Experiments I and II are summarized in Tables 2 and 3, respectively.

### 3.3. Results of Classifiers

In this study, we presented experimental results obtained from the M-SVMs and ANFIS algorithms, which are three class nonlinearly separable problems. In case of experiments, optimal *C* value was chosen as 10^7^ and regularization parameter (Lambda) was 10^−7^ for M-SVMs. However, for ANFIS predictions, maximum number of epochs was chosen as 10. It is seen that this is enough to allow the system converge to a final value. The learning rate was chosen as 0.0, and examined membership functions for ANFIS predictions are Gaussian combination, Gaussian curve, generalized bell-shaped, pi-shaped, sigmoidally shaped, trapezoidal-shaped, and triangular-shaped membership functions. It is observed that the best prediction results in the same conditions obtained by generalized bell-shaped membership function.

For each experiment, the classification performance was evaluated in terms of sensitivity defined as 100 × TP/(TP + FN), positive predictive value (PPV) defined as 100 × TP/(TP + FP), and accuracy 100 × (TP + TN)/(TP + TN + FP + FN), where TP, TN, FP, and FN are the numbers of true positive, true negative, false positive, and false negative classified segment parts, respectively.

Tables 4 and 5 show the classifiers performances of Experiments I and II for snore, breathing, and silent segment parts. Herein, all segments have different number of segment parts (each part having 3200 samples); owing to the segment time interval, the total numbers of segment parts in training and test data sets are different from each other.

**Figure 6:**
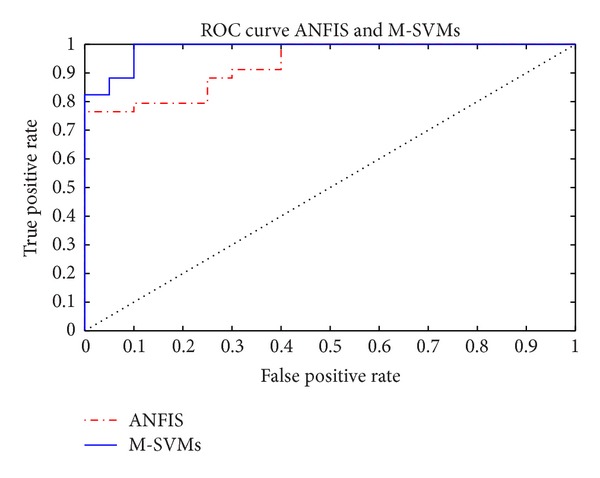
Receiver operating characteristics (ROC) curve of ANFIS and M-SVMs.

Tables 4 and 5 represent information about percentages of accuracy results of training and test datasets. It can be seen that M-SVMs give a good performance concerning both training and test procedures in Experiments I and II. The best detection performance was achieved in Experiment I where both training and test datasets have different time intervals of same subjects' recordings (Table 4). In Experiment II (Table 5), the SVMs test accuracy was dropped by 6.96% (from 91.61% to 84.65%), while in ANFIS test accuracy was dropped by 6.2% (from 86.75% to 80.55%). Herein, training and test datasets were obtained from different individuals. Additionally, looking at the testing sensitivities of snore and silent segment parts for SVMs was close to training performance, especially in Experiment I. However, breathing segment parts' sensitivity results are worse than others because breathing segment beginning and ending parts are close to snore or silent parts. That is, the entropy and LLE values are similar to others on segment beginning and ending parts. So, classifier is having difficulty in classifying breathing segment parts. The similar results were again obtained by ANFIS, but the accuracy was not feasiblly good as SVMs.  Figure 6 gives information about ROC analyses of methods.

## 4. Discussion and Conclusion

It can be seen from the previously performed studies that some different features, obtained from the analysis of sound signals in spectral and temporal domains, were used for examining the SRSs. In majority of these works, the energy of sounds, zero-crossing rates, MFCCs, and formant frequencies, and so forth, were estimated, and linear frequency analyses such as LPC and Fourier transform were examined [17–20]. SRSs cannot be adequately examined by linearly based methods since they have complex nature [21, 22]. The nonlinear analysis can bring out the SRSs' characteristics related to irregular behaviour.

In this study, the significant information to understanding of chaotic behaviour of SRSs and useful features to classifying the SRSs was expected from the nonlinear measures. For this goal, the LLE and entropy measures were used for classifying SRSs into three classes. The LLE and entropy values of SRSs segments in Experiment II were compared with each other by using unpaired Student's *t*-test with level of significance *P* < 0.001 (with Bonferroni correction), statistically. The LLE calculated for all SRSs segments have a positive value, and this result can be accepted as an evidence of chaotic behaviour. It can be recognized that from experiments, that the LLE values of breathing and silent groups were found higher, and entropy values of these two groups were found lower than snore group, significantly. However, for breathing group, the LLE value is lower and the entropy value is higher than silent group, significantly. So, the results have shown that while the LLE values tend to decrease, the entropy values tend to increase from silent to snore segments. This decrement for LLE indicates that dynamics of snore segment have low-level complex behaviour with respect to breathing and silent segments. The reason for this can be interpreted depending on frequency characteristics of SRSs [21]. SRSs segments have different frequency and amplitude spectrums. Snore segments generally consist of simple and complex waveforms and have irregular appearance [21]. They have some high and low amplitude peaks for different frequency range in domain [21]. However, breathing and silent segments show noise-like characteristics which have many similar peak values in a wide frequency band [17, 20]. These segments generally appear as intertwined with each other, with some background noises especially originated from ambient recordings [17, 20]. Because of giving a measure of predictability and complexity of sounds, the increments of LLE show that the predictability of system decreases from snore to silent segment.

The entropy values may be interpreted as the average amount of new information obtained by making the measurement in information theory sense [35]. According to entropy values, it can be said that the information expected from snore segment is greater than breathing and silent. This result can be evaluated from a different perspective; according to Williams [35], high entropy is interpreted as high accuracy in the data, and it shows that the state of system can be determined with great accuracy. The LLE and entropy measurements produced significant values for differentiating these segments if these results were taken into account.

The structure of SRSs shows variations such as silent, breathing, and snore segments, so the degree of chaotic behaviour of SRSs can vary based on these segments. According to LLE values, the complexity of snore segment has lower level than others, and it can be said that, considering entropy values, the state of system can be defined as more accurate than breathing and silent segments. It is found that the complexity increases and amount of new information gained from measurement decreases from snore to silent segments. Since the silent segment has not any information because of noisy characteristics, SRSs are actually produced during breathing process, and therefore the dynamics of these signals depend on the breathing cycle. Cervantes and Femat [60] have studied the phenomenon of breathing in a class of piecewise continuous systems by using a model of linear-driven switched system. It is seen that chaotic and regular phases of breathing evolve irregularly in their system; however, their average behaviour is surprisingly regular depending on bifurcation parameter. Therefore, they have shown that breathing phenomenon occurs the similar structural characteristics with intermittency. If this result is taken into account, it can be said that SRSs show intermittency behaviour since they have different forms of chaotic dynamics.

Different from previous studies [17–20], although there are many classifier methods, multiclass SVMs and ANFIS were selected in this study. First, data was trained by one-against-all SVMs and ANFIS which provide a feasible way to categorize and classify SRSs in experiments. While experiments were realized, different kernel functions such as polynomial, gauss, RBF gauss. were used for obtaining best accuracy results. It has been shown that when gauss-based kernel functions are used for both classifiers, the system accuracy was the best.

The previous studies have shown that classification accuracies of them have varied values depending on their experimental procedures [17–20]. Duckitt et al. [17] obtained snoring detection sensitivity as 89% with training and test data from same patients and 82.2% with training and test data sets from different patients. In Cavusoglu et al.'s study [18], their sensitivities for snore detection were 97.3% with only simple snorers and 90.2% with both simple snorers and OSA patients. For OSA patients, their sensitivity was 86.8%. These results were found when training and test data sets were formed of different individuals [18]. Karunejeewa et al.'s method [19] provided 90.74% total sensitivity for snoring, breathing, and silence classification. They also used three different noise reduction techniques and obtained total maximum sensitivity of 96.78% with proper choices of noise reduction technique and features. Their test set contained different subjects' recordings than those used in training set [19]. Yadollahi and Moussavi [20] used both tracheal and ambient sounds gathered from different body and neck positions to classify them into breath and snore groups. Their results were obtained by using different subjects' samples as training and test data sets [20], and their total accuracies were found as 95.7% and 93.2% for tracheal and ambient recordings, respectively.  Table 6 gives information about previous studies and our study accuracy results.

In this paper, it is seen that SRSs have nonlinear characteristics, which carry any information about snore, breathing, and silent segments of SRSs. With the help of this information, these segments can be differentiated into different groups. Two experimental procedures were implemented so that training and test data sets were formed in two ways: same (for Experiment I) or different (for Experiment II) subjects' data. The total classifiers performances of Experiment II (84.65% for M-SVMs and 80.55% for ANFIS) were found lower than Experiment I (91.61% for M-SVMs and 86.75% for ANFIS). In previous studies, they generally focused on estimating snore detection sensitivities (Table 6). However, snore detection sensitivities were found as 91.49% for M-SVMs, 79.31% for ANFIS in Experiment I and 87.58% for M-SVMs, 73.28% for ANFIS in Experiment II. The results show that proposed methods especially M-SVMs classification accuracy is as good as previous studies.

Table 6 shows that previous studies have generally focused on linear analysis of SRSs segments and have tried to detect snore segments. In fact, time domain linear features are important for extracting any information from physiological signals. It can be seen from these studies that their results were good enough to classify and differentiate the SRSs. However, keeping in mind the chaotic behaviour of these sounds is also considerable. Experimental results have shown that the LLE and entropy can be important features for clinical diagnosis systems.

## Figures and Tables

**Table 1:** Information of patients.

Subject no.	AHI	BMI	Age	Gender
1	1.27	26.03	39	F
2	9.2	27.28	51	M
3	14.3	24.38	40	M
4	14.7	27.36	42	M
5	17.7	27.46	44	M
6	19	32.47	55	F
7	20.3	28.41	36	M
8	22	26.25	55	M
9	24	26.78	25	M
10	24.6	33.31	59	F
11	24.7	26.1	67	M
12	29.86	29.67	48	M

**Table 2:** The details of training and test data sets in Experiment I.

Subject no.	Training			Test		
	12 subjects (first half interval)			12 subjects (rest interval)		
	Snore	Breathing	Silent	Snore	Breathing	Silent
1	25	23	16	25	23	15
2	15	15	15	15	15	15
3	15	15	15	15	15	15
4	15	15	15	15	15	15
5	25	25	8	25	25	8
6	39	25	11	39	25	10
7	25	15	25	25	15	25
8	25	25	25	25	25	25
9	25	15	25	25	15	25
10	25	25	10	25	25	10
11	0	10	7	0	10	6
12	25	25	8	25	25	7

Total	259	233	180	259	233	176

Total	672			668		

**Table 3:** The details of training and test data sets in Experiment II.

Training				Test			
Subject no.	Snore	Breathing	Silent	Subject no.	Snore	Breathing	Silent
2	30	30	30	1	50	46	31
3	30	30	30	5	50	50	16
4	30	30	30	6	78	50	21
9	50	30	50	7	50	30	50
10	50	50	20	8	50	50	50
11	0	20	13	12	50	50	15
Total	190	190	173	Total	328	276	183

Total	553			Total	787		

**Table:** (a)

	Number of segment parts	Snore	Breathing	Silence	Sensitivity (%)	PPV (%)	Total accuracy (%)
M-SVMs training							
Snore	1260	1190	10	60	94.44	100	**92.79**
Breathing	746	0	546	200	73.19	94.63	
Silence	2031	0	21	2010	98.97	88.55	

M-SVMs test							
Snore	1175	1075	42	58	91.49	97.64	**91.61**
Breathing	746	10	457	279	61.26	85.74	
Silence	3313	16	34	3263	98.49	90.64	

**Table:** (b)

	Number of segment parts	Snore	Breathing	Silence	Sensitivity (%)	PPV (%)	Total accuracy (%)
ANFIS training							
Snore	1260	1150	91	19	91.26	96.88	**86.97**
Breathing	746	32	597	117	80.02	62.84	
Silence	2031	5	262	1764	86.86	92.84	

ANFIS test							
Snore	1175	932	202	41	79.31	94.81	**86.75**
Breathing	746	35	426	285	57.10	57.41	
Silence	3313	16	114	3183	96.07	90.70	

**Table:** (a)

	Number of segment parts	Snore	Breathing	Silence	Sensitivity (%)	PPV (%)	Total accuracy (%)
M-SVMs training							
Snore	1156	1097	42	17	94.90	96.65	**90.07**
Breathing	721	32	525	164	72.82	82.55	
Silence	1445	6	69	1370	94.81	88.33	

M-SVMs test							
Snore	1280	1121	135	24	87.58	85.57	**84.65**
Breathing	772	175	523	74	67.75	60.32	
Silence	2058	14	209	1835	89.16	94.93	

**Table:** (b)

	Number of segment parts	Snore	Breathing	Silence	Sensitivity (%)	PPV (%)	Total accuracy (%)
ANFIS training							
Snore	1156	971	162	23	83.99	96.14	**84.61**
Breathing	721	39	423	259	58.67	69.00	
Silence	1445	0	28	1417	98.06	83.40	

ANFIS test							
Snore	1280	938	303	39	73.28	87.91	**80.55**
Breathing	772	128	414	230	53.62	50.79	
Silence	2058	1	98	1959	95.19	87.92	

**Table 6:** Snore sound classification studies and their accuracy results.

Study	Duckitt et al. [17]	Cavusoglu et al. [18]	Karunajeewa et al. [19]	Yadollahi and Moussavi [20]	Our method
Sound types	Ambient sound	Ambient sound	Ambient sound	Ambient and tracheal sound	Ambient sound
Classes	Snoring and other sounds (silence, breathing, and other types of sounds); snore detection	Snore/nonsnore	Snore, breathing, and silence	Snore and breathing	Snore, breathing, and silence
Features	39-dimensional feature vector of energy and MFCC	Spectral energy distributions	Zero-crossings and signal's energy	Zero-crossings signal's energy, and first formant	The largest Lyapunov exponent (LLE) and entropy
Classifier	HMM	Linear regression	Minimum-probability-of-error decision rule	FLD	M-SVMs and ANFIS
Accuracy	**82–89%** snore sensitivity	**86.8%** snore sensitivity	**90.74%** total sensitivity	**93.2%** for ambient sound total accuracy	In Exp. I: **91.61%** (SVMs), 86.75% (ANFIS) total accuracies; and **91.49%** (SVMs), 79.31% (ANFIS) snore sensitivities
